# Imidazolium-Based Ionic Liquid Electrolytes for Fluoride Ion Batteries

**DOI:** 10.1021/acsenergylett.4c02663

**Published:** 2024-11-27

**Authors:** Omar Alshangiti, Giulia Galatolo, Camilla Di Mino, Thomas F. Headen, Jacob Christianson, Simone Merotto, Gregory J. Rees, Yoan Delavoux, Małgorzata Swadźba-Kwaśny, Mauro Pasta

**Affiliations:** † Department of Materials, University of Oxford, Oxford OX1 3PH, United Kingdom; ‡ ISIS Neutron and Muon Source, Science and Technology Facilities Council, Rutherford Appleton Laboratory, Didcot OX11 0QX, United Kingdom; § Department of Chemistry, University of Oxford, Oxford OX1 3TA, United Kingdom; ∥ The QUILL Research Centre, School of Chemistry and Chemical Engineering, Queen’s University of Belfast, Belfast, BT9 5AG, Northern Ireland, United Kingdom

## Abstract

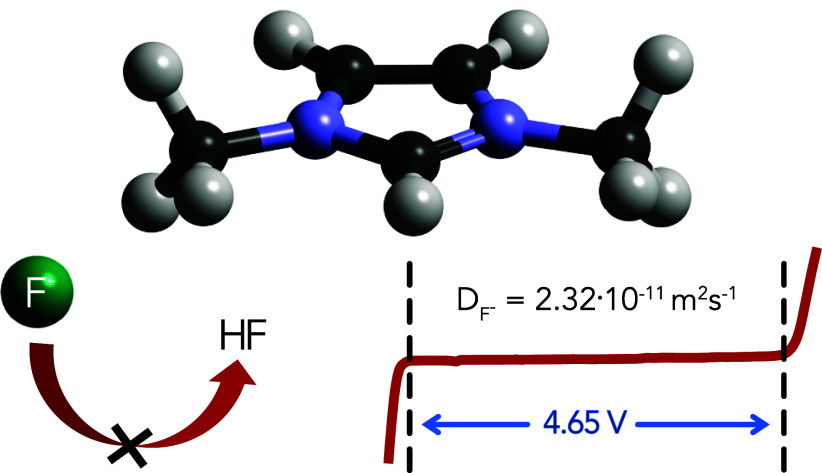

The fluoride-ion battery (FIB) is a post-lithium anionic battery that utilizes the fluoride-ion shuttle, achieving high theoretical energy densities of up to 1393 Wh L^–1^ without relying on critical minerals. However, developing liquid electrolytes for FIBs has proven arduous due to the low solubility of fluoride salts and the chemical reactivity of the fluoride ion. By introducing a chemically stable electrolyte based on 1,3-dimethylimidazolium [MMIm] bis(trifluoromethanesulfonyl)imide [TFSI] and tetramethylammonium fluoride (TMAF), we achieve an electrochemical stability window (ESW) of 4.65 V, ionic conductivity of 9.53 mS cm^*–*1^, and a solubility of 0.67 m. The origin of this high solubility and the solvation structure were investigated using NMR spectroscopy and neutron total scattering, showing a fluoride solvation driven by strong electrostatic interactions and weak hydrogen bonding without covalent H–F character. This indicates the chemical stability of 1,3-dimethylimidazolium toward the fluoride ion and its potential as an electrolyte for high-voltage FIBs.

The fluoride-ion battery (FIB) is an emerging postl-ithium-ion technology based on the shuttling of the fluoride ion, with theoritical energy densities of up to 1393 Wh L^*–*1^ (588 Wh kg^*–*1^).^1^ However, designing electrolytes for FIBs has proven challenging due to the insolubility of fluoride salts in aprotic organic solvents (e.g., carbonates, ethers, sulfolane), which are known for their wide electrochemical stability windows (ESW). Additionally, the fluoride ion tends to deprotonate even slightly acidic solvents, such as acetonitrile, leading to the formation of corrosive HF.^1,2^ This combination of low solubility and chemical instability has limited the range of potential FIB solvents and required novel, yet so far unsatisfactory, electrolyte systems.

Three main families of liquid electrolytes have been investigated in the FIB field: anion acceptors, binary aprotic electrolytes and electrolytes utilizing protic solvents. One widely reported electrolyte for FIBs utilized boron-based anion acceptors to facilitate the dissolution of cesium fluoride (CsF) in tetraglyme ethers.^3^ The formation of a soluble fluoroborate complex increased the CsF solubility to around 0.5 mol L^–1^, enabling the fluoride shuttling and conversion. However, the strong binding energy and the necessity for high concentrations of the anion acceptor led to active material dissolution and rapid capacity fading.^4,5^ This issue was partially addressed by using anion acceptors with milder fluoride binding energy, resulting in faster electrode/electrolyte interface desolvation and higher ionic conductivity of 2.40 mS cm^–1^, although the ESW remained around 3 V.^6^

Few binary electrolytes have been reported for FIBs. A notable system employed a branched quaternary ammonium fluoride salt dissolved in bis(2,2,2-trifluoroethyl) ether (BTFE), achieving an ESW of around 4.1 V and enabling some cycling of CuF_2_ againts a lanthanum anode.^7^ The selection of BTFE was due to its stability toward HF formation, though some HF formation still persisted.^2^ Other reported electrolytes include aqueous,^8−10^ alcohol-based,^11−14^ and deep eutectic systems,^15^ but all with ESWs of around 3 V or less.

Ionic liquids (ILs) are considered excellent solvents for battery electrolytes due to their low vapor pressure, high thermal and chemical stability, and wide ESW.^16^ To the best of our knowledge, only two ionic liquid FIB electrolytes have been reported. Okazaki et al. used an ammonium-based ionic liquid, achieving a solubility of 0.35 mol L^–1^, an ionic conductivity of 2.5 mS cm^–1^, and an ESW of 0.70 V.^17^ Another report demonstrated the use of fluorohydrogenate ionic liquids (FHIL), but without evidence of (electro)chemical stability.^18^ Among ionic liquids, imidazolium-based ILs have polarities similar to acetonitrile and short chain alcohols, while still maintaining the advantages of wide ESW and low vapor pressure.^16^ Herein, 1,3-dimethylimidazolium [MMIm] bis(trifluoromethanesulfonyl)imide [TFSI] was selected as a model imidazolium electrolyte solvent, with the rationale of avoiding imidazoliums with β-hydrogens which are known to undergo Hoffman elimination.^1^ [MMIm][TFSI] was synthesized via a chloride-free route through alkylation of 1-methylimidazole with methyl bis(trifluoromethanesulfonyl)imide (Scheme 1).

**Scheme 1:**
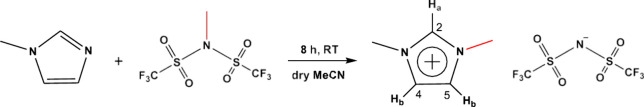
Synthesis of 1,3-Dimethylimidazolium Bis(trifluoromethanesulfonyl)imide, [MMIm][TFSI], through One-Step, Chloride-Free Alkylayion

The C2 hydrogen in imidazolium is known to be relatively acidic and prone to forming the *N*-heterocyclic carbene (NHC) upon deprotonation, as evident by its exchange with deuterium.^19^ This deprotonation, however, has been previously reported with relatively strong bases such as 3-hydroxyquinuclidine (3-HQD) and 1,4-diazabicyclo[2.2.2]octane (DABCO),^20^ whereas the fluoride ion (p*K*_a_ 3.2)^21^ is a much weaker base than both DABCO and 3-HQD (p*K*_a_ 8–9).^20^ To test the chemical stability of imidazolium toward the fluoride ion, [MMIm][TFSI] was studied using ^13^C NMR spectroscopy and electron paramagnetic resonance (EPR) spectroscopy, to detect any NHC species that may have formed. Upon the addition of TMAF, the only new ^13^C NMR signal at 54.7 ppm was due to the TMAF methyl groups (Figure 1a). The signals at 35.2 and 123.1 ppm were attributed to the imidazolium N–CH_3_ and the two equivelent C4 and C5 carbons (Scheme 1), respectively, and the 119.68 ppm quartet to the CF_3_ in [TFSI]^*–*^ (Supporting Information Figure S8). NHCs are known to show ^13^C NMR signals in the region of 206–220 ppm.^22^ The lack of such signal (Figure 1a, inset) suggests the absence of carbene formation and thus the stability of [MMIm]^+^ toward fluoride deprotonation. Furthermore, EPR spectroscopy performed at −170 °C showed only one broad background signal, which persisted after the addition of TMAF (Figure 1b). Given that imidazolium NHCs tend to be in the singlet state due to their orbital geometry,^23^ EPR was performed at RT to promote the excitation of any singlet NHC to the triplet state,^24^ but an identical EPR spectrum was obtained, further indicating the absence of NHCs. Additionally, DFT calculations show a higher ground-state energy of the NHC compared to that of [MMIm][TFSI], indicating that deprotonation is energetically unfavorable (Figure S5).

**Figure 1:**
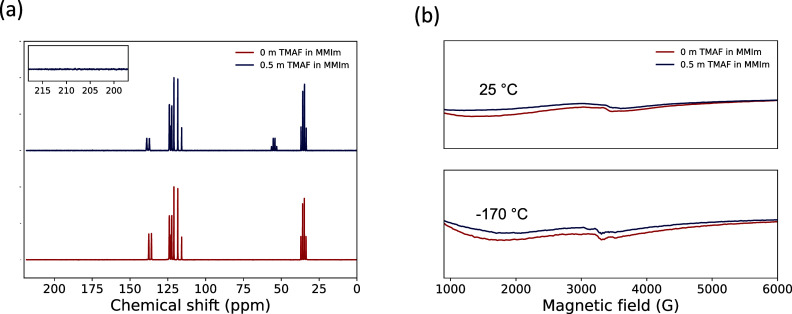
Chemical stability of [MMIm][TFSI] against fluoride deprotonation and NHC formation. ^13^C NMR (a) and EPR (b) spectroscopies for [MMIm][TFSI] and 0.5 *m* TMAF in [MMIm][TFSI].

Given this evidence against C–H deprotonation by the fluoride, the role of the proton in fluoride solvation was examined using ^1^H NMR spectroscopy and neutron total scattering. ^1^H NMR spectra suggested preferential fluoride solvation by the C2 proton (*H*_a_), as evidenced by the more pronounced change in chemical shift compared to the C4/5 protons (*H*_b_) upon increased fluoride concentration (Figure 2a). NMR spectroscopy, however, is not sufficient in determining the specific positions and distances of the solvating motifs. Therefore, a neutron scattering study with H/D isotopic contrast was carried out to elucidate the liquid structure of the electrolyte (Figures S3 and S4). Reduced data were analyzed via the empirical potential structure refinement (EPSR) method (Figures S1 and S2 and Table S2 for EPSR input parameters and molecular geometries).^25^ The CH···F partial radial distribution function, *g*(*r*), can be used to exclude the presence of a short hydrogen bonding interaction with covalent character.^26^ Such hydrogen to chemical bond crossover typically occurs at distances ∼1 Å.^26^ The H···F *g*(*r*) presents an intense peak at 2.30 Å, with a second peak at ∼5.9 Å (Figure 2b), indicating the presence of weak CH···F bonding, with no evidence of shorter distances indicative of covalent character. The distance-angle map shows an intense peak centered at 130°, with the second closest fluoride ion at 40° (Figure 2c), confirming the weak and nondirectional C–H···F hydrogen bonding.^27^ The spatial density function (SDF) from the imidazolium center-of-mass (CoM) (Figure 2d) shows that the average position of the fluoride ion lays between the imidazolium C–H and the methyl groups, with stronger interaction with the imidazolium C–H as evidenced by the shorter distance.

**Figure 2:**
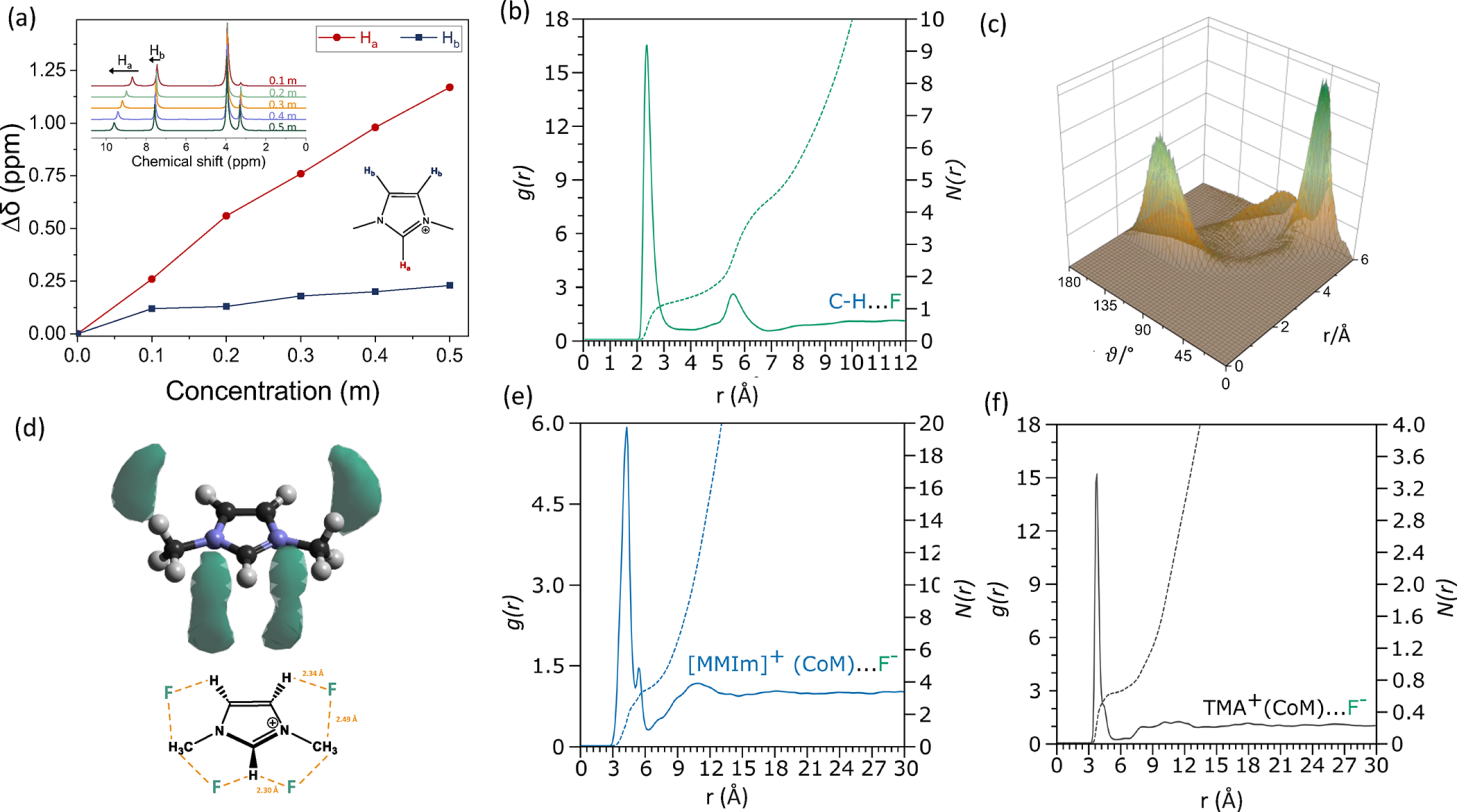
Solvation structure of the fluoride ion in [MMIm][TFSI]. ^1^H NMR at variable TMAF concentrations showing the change in the proton chemical shift (a). Partial radial distribution function, *g*(*r*), and coordination number, *N*(*r*), for CH···F (b). Distance–angle map for CH···F (c). Spatial density function (SDF) for the fluoride ion around [MMIm]^+^ (d). Partial radial distribution function, *g*(*r*), and coordination number, *N*(*r*), for MMIm···F (e) and TMA···F (f).

The partial radial distribution functions, *g*(*r*), and coordination numbers, *n*(*r*), plotted for the [MMIm]^+^ CoM and F^–^ (Figure 2e) and for the [TMA]^+^ CoM and F^–^ (Figure 2f) show high intensity peaks for both MMIm···F (4.65 Å) and TMA···F (3.65 Å). This indicates a highly localized interaction between the two cations and the fluoride. From integrating the two curves up to the first *g*(*r*) minima (6.75 and 5.45 Å, for [MMIm]^+^ and [TMA]^+^, respectively), the first solvation shell of the fluoride ion is found to contain ca. 5 [MMIm]^+^ and 0.6 [TMA]^+^ cations. This imidazolium-dominated solvation shell of the fluoride, in addition to the absence of low-*Q* signal in the neutron scattering data, and the low simulated average TMA···F coordination number (Figure 2f) suggests the absence of [TMA]^+^ and F^*–*^-ion clustering. Good solubility of TMAF in [MMIm][TFSI] can be attributed to the combination of strong Coulombic interactions and weak hydrogen bonding. In other words, this electrolyte is a mixture of four statistically distributed ions: MMIm^+^, TMA^+^, F^–^, and TFSI^+^.^28^ This resulted in TMAF solubility of 0.67 m (Figure S6), higher than previously reported aprotic FIB solvents.^7^

The absence of carbene, potentially arising from deprotonation of the imidazolium cation, combined with relatively weak hydrogen bond between the most acidic proton and the fluoride ion, confirm that 1,3-dimethylimidazolium is intrinsically stable toward the fluoride ion. Nevertheless, traces of hydrogen bifluoride, [HF_2_]^−^ have been observed in ^19^F NMR spectrum of the TMAF solution in [MMIm][TFSI] (Figures S7 and S10). Despite meticulous drying under ultrahigh vacuum, levels of moisture in [MMIm][TFSI] and TMAF remained at 100–300 ppm, acting as the source of protons for the [HF_2_]^−^ anion.

Given the high fluoride solubility and chemical stability of [MMIm][TFSI], the electrolyte transport and electrochemical properties where subsequently examined. The ionic conductivity was measured using electrochemical impedance spectroscopy (EIS) to be 9.53 mS cm^*–*1^ for the 0.1 *m* electrolyte, decreasing to 6.16 mS cm^*–*1^ for the 0.5 *m* (Figure 3a, Figure S9). This trend is in agreement with previously reported ionic conductivities of ionic liquids, decreasing monotonically with increasing salt concentration due to the increased viscosity.^29,30^ The fluoride diffusivity, determined by pulse-field gradient (PFG) NMR spectroscopy, also matched this trend, with self-diffusion coefficients ranging between 2.32 × 10^–11^ and 1.36 × 10^–11^ m^2^ s^–1^ for the 0.1 and 0.5 *m*, respectively (Figure 3b). The 0.1 *m* solution of TMAF in [MMIm][TFSI] was thus used for further testing due to its high ionic conductivity and diffusivity. The ESW was measured using linear sweep voltammetry (LSV) to be 4.65 V (Figure 3c) using a 100 μA cm^–2^ current density cutoff. This, to the best of our knowledge, is the highest ESW reported thus far for a FIB electrolyte. Furthermore, the thermal stability of TMAF in [MMIm][TFSI] was demonstrated using thermogravimetric analysis (TGA), showing excellent thermal stability up to 400 °C (Figure 3d). This thermal stability, associated with [MMIm][TFSI], was maintained even in the presence of TMAF, which exhibits a much lower decomposition temperature of 170 °C.^31^ This further supports the conclusion that the solution of TMAF in [MMIm][TFSI] is simply a mixture of four equally dispersed ions. Differential scanning calorimetry (DSC) analysis corroborated that high thermal stability by the lack of phase changes at higher temperatures (Figure 3e). Finally, cyclic voltammetry showed that the achieved diffusivity and ionic conductivity were sufficient to cycle both high- and low-voltage metal fluorides such as AgF_2_ and MnF_2_ (Figure 3f).

**Figure 3:**
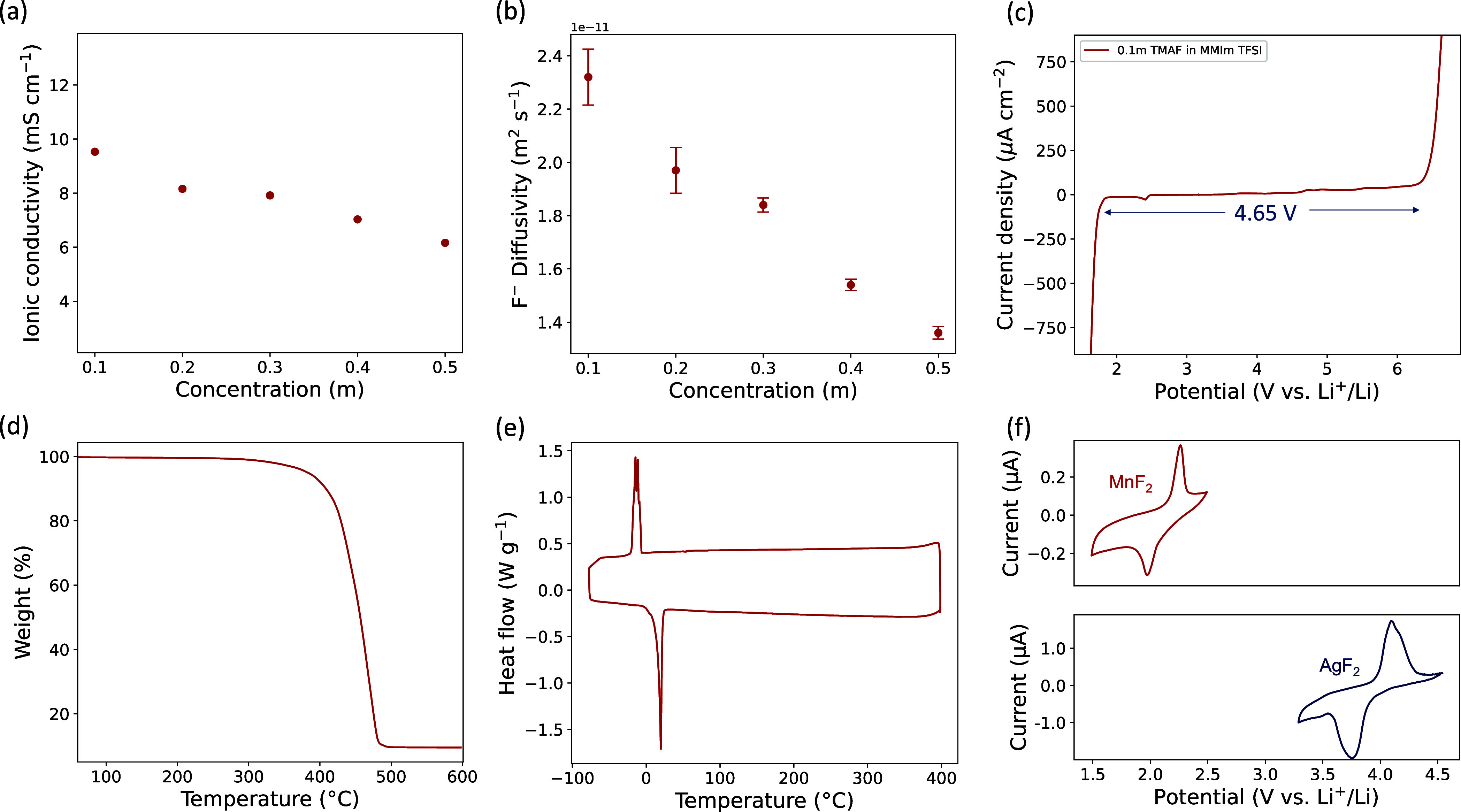
Electrochemical, transport, and thermal properties of 0.1 *m* solution of TMAF in [MMIm][TFSI]. Electrolchemical stability window (a). Fluoride-ion diffusion coefficient (b) and ionic conductivity (c) at variable TMAF concentrations. Thermogravimetric analysis (TGA) (d) and differential scanning calorimetry (DSC) (e) showing thermal stability up to 400 °C.

In conclusion, [MMIm][TFSI] was shown to be an effective solvent for FIB electrolytes, with exceptional electrochemical stability, ionic conductivity and chemical inertness toward the fluoride ion, while maintaining adequate diffusivity to allow for RT cycling of metal fluorides. Compared to other aprotic organic solvents, [MMIm][TFSI] exhibited higher fluoride solubility. Neutron total scattering showed the role of weak hydrogen-bonding and electrostatic interactions in facilitating the solvation of the fluoride ion, without proton abstraction from the imidazolium ring, thereby avoiding autocatalytic decomposition of the electrolyte. These results pave the way for further exploration and design of ionic liquid electrolytes to enable RT high-voltage FIBs.

## Data Availability

Unprocessed neutron total scattering data can be found at 10.5286/ISIS.E.RB2410443-2.

## Supporting Information Available

Materials and experimental methods for electrochemical testing, NMR spectra, and neutron total scattering theory and input parameters; supplementary figures including parametrized atom types, total structure factors, and total pair distribution functions against EPSR fits, and ^19^F NMR specra (PDF)
