# Supplementary material for: Imidazolium-Based Ionic Liquid Electrolytes for Fluoride Ion Batteries
Source: ACS Energy Lett. 2024 Nov 27;9(12):6104–8. doi: 10.1021/acsenergylett.4c02663 (PMC11651116; doi:10.1021/acsenergylett.4c02663)
Supplement: Supplementary file 1 — nz4c02663_si_001.pdf [file nz4c02663_si_001.pdf]

Supporting Information

Imidazolium-based ionic liquid electrolytes for fluoride ion batteries

Omar Alshangiti,[†,‖] Giulia Galatolo,[†,‖] Camilla Di Mino,[†,‖] Thomas F. Headen,[‡] Jacob Christianson,[¶] Simone Merotto,[†] Gregory J. Rees,[†] Yoan Delavoux ,[§] Małgorzata Swadźba-Kwaśny,[§] and Mauro Pasta[*,†]

†*Department of Materials, University of Oxford, Oxford, OX1 3PH, UK*

‡*ISIS Neutron and Muon Source, Science and Technology Facilities Council, Rutherford Appleton Laboratory, Didcot, OX11 0QX, UK*

¶*Department of Chemistry, University of Oxford, Oxford, OX1 3TA, UK*

§*School of Chemistry and Chemical Engineering, Queen's University of Belfast, Belfast, BT9 5AG, Northern Ireland, UK*

‖*Contributed equally to this work*

E-mail: mauro.pasta@materials.ox.ac.uk

Methods

Synthesis of [MMIm][TFSI]

1,3-dimethylimidazolium [MMIm] bis(trifluoromethanesulfonyl)imide [TFSI] was synthesized using direct methylation of 1-methylimidazole (TCI) by methyl bis(trifluoromethanesulfonyl)imide (ABCR). In a typical experiment, 1-methylimidazole, freshly distilled (40 °C, 1 mbar) was collected in a round-bottomed flask (50 ml), under argon, and transferred into an argon-filled

MBraun glovebox (<0.3 ppm of O_2 and H_2O). Methyl bis(trifluoromethanesulfonyl)imide was used as received after being flushed under argon for 30 min prior to transfer in the glovebox. In the glovebox, dry degassed acetonitrile (10 ml) was added to the round-bottomed flask containing 1-methylimidazole (2.8 g, 1 mol eq.), along with a stirring bar. Methyl bis(trifluoromethanesulfonyl)imide, MeTFSI (10 g, 1 mol eq.) and dry degassed acetonitrile (10 ml) were placed in a pressure-equalizing dropping funnel (50 ml), which was then closed with a stopper and mounted on the flask containing 1-methylimidazole solution. The solution of MeTFSI was added dropwise to the vigorously stirred 1-methylimidazole solution, at a slow pace to control the evolved heat. After the addition, the reaction mixture was allowed to react (ambient temperature, 8 h), until 1-methylimidazole signals were invisible in ^{1}H NMR spectra. Subsequently, the flask containing the reaction mixture was removed from the glovebox and solvent was removed under reduced pressure (rotary evaporator, 50 °C, 132 mbar). Then, the ionic liquid was stirred under high vacuum (60 °C, $<10^{-3}$ mbar, 600 rpm, overnight) and subsequently under ultra-high vacuum (60 °C, $<10^{-7}$ mbar, 600 rpm, 8 h). ^{1}H NMR (400 MHz, DMSO-d_6) 8.4 ppm (s, 1 H), 7.3 ppm (s, 2 H), 3.8 ppm (s, 6 H), ^{13}C NMR (101 MHz, DMSO-d_6) 135.8 ppm, 122.3 ppm, 118.8 ppm (q, CF_3), 34.5 ppm, ^{19}F NMR (376 MHz, DMSO-d_6) -80.93 ppm. The deuteration processes was carried out using a probe sonication (10 minutes, RT) of 1 mL of [MMIm][TFSI] added to 9 mL of 0.05 m sodium bicarbonate in D_2O to obtain d_1-1,3-dimethylimidazolium-bis(trifluoromethanesulfonyl)imide and the purity of the d_1 product confirmed by the 1H NMR and the disappearance of the 8.4 ppm signal.

Synthesis of TMAF

Tetramethylammonium fluoride (TMAF) was synthesized starting from KF and $TMABF_4$ (Sigma Aldrich), dissolved in methanol and followed by the filtration of the KBF_4 precipitate under argon and the removal of the solvent in-vacuo using a solvent trap. TMAF was then dried under high vacuum turbopump ($<10^{-3}$ mbar) at 150 °C for 3 days. The synthesis was

carried out due to the less hygroscopic nature of KF and $TMABF_4$ compared to TMAF, with the objective of achieving TMAF with lower water content under meticulously drying conditions.

Karl-Fischer measurement

The water content was measured using a Mettler Toledo C30S coulometric Karl-Fischer titrator and a Coulomat AG hydranal (purchased from Honeywell). The water level (in ppm) of the neat solvent was measured, followed by a measurement of the salt-containing electrolyte to determine the water content of the TMAF salt.

Linear sweep and cyclic voltammetry

LSV was performed at 1 $mV s^{-1}$ in a 3-electrode custom-made polytetrafluoroethylene (PTFE) cell with glassy carbon working electrode, Pt wire counter electrode, and Ag wire pseudo-reference calibrated using ferrocene. CV was performed using the corresponding metal casting as working electrode, Pt wire as counter electrode, and Ag wire as reference electrode. The casting was prepared by solventless mixing of the corresponding ratios of active material, conductive carbon fiber, and PTFE powder. Biologic VMP3 potentiostats were used for cycling. The ESW was defined based on a cutoff of 100 uA/cm^2.

Nuclear Magnetic Resonance (NMR)

Diffusion coefficients were obtained using pulsed field gradient (PFG) NMR by Bruker Avance III HD spectrometer equipped with a 5 mm single-axis diffusion probe with exchangeable ceramic heads (9.45 T (ν_0(1H) = 400.19 MHz, $\nu_0(^{19}F)$ = 376.51, and $\nu_0(^{133}Cs)$ = 52.49 MHz). All samples were run in air-tight J-Young NMR tube. Stimulated echo pulse sequence was used for all PFG experiments (δ 1 - 1.5 ms, Δ 10-30 ms, and the gradient amplitude 0.1 -

24 T/m in 36 steps). All data was fitted to the Stejskal-Tanner equation:

$$f(x) = I_0 e^{-\gamma^2 g^2 \delta^2 \frac{(\Delta - \delta)}{3} D} \tag{1}$$

With R^2 value 0.999, errors of $< 1\%$ and normal distribution of the residuals (>0.5 Shapiro Wilko score).

Ionic conductivity measurements

Electrochemical impedance spectroscopy (EIS) was performed at 30 °C at frequency range 1 mHz - 1 MHz, using a custom-made Polyether ether ketone (PEEK) cell with stainless steel blocking electrodes with surface area of 0.494 cm^2 and diffusion length of 0.642 cm.

Density Functional Theory (DFT) calculations

The DFT calculation were carried with Gaussian16 using B3LYP with basis set 6-311G++-(p,d) in vacuum. The diffuse basis set (++) was used to take into account the effect of polarization induced by the presence of the positive charge on imidazolium. Charges were added or removed from the atoms to create the corresponding fragments to simulate MMIm and the deprotonated MMIm (the carbene), respectively. A first geometric optimization calculation was run with a tight energy convergence criteria of 10^{-12} Ry followed by ground-state calculation to compute the two corresponding ground-state energies.

Neutron Total Scattering

Neutron total scattering is a very powerful tool for the study of disordered materials due to the wide Q range accessible, that translates to a very fine real spatial resolution, and to the sensitivity to light elements such as hydrogen (H) and deuterium (D). By exploiting the different magnitude and sign of the H and D scattering lengths ($b_H = -3.74 fm; b_D = +6.67 fm$), we can measure different total structure factors $F_i(Q)$, that are the weighted sum

of the partial structure factors arising from each pair of atoms α and β:

$$F(Q) = \sum_{\alpha,\beta\geq\alpha}(2-\delta_{\alpha\beta})c_\alpha c_\beta b_\alpha b_\beta(S_{\alpha\beta}(Q)-1) \quad (2)$$

where Q is the momentum change vector of the neutron of wavelength λ defined as $Q = 4\pi\frac{sin\theta}{\lambda}$; c_α, c_β are the atomic fractions of species α and β respectively and b_α, b_β their relative neutron scattering lengths; and $S_{\alpha\beta}$(Q) is the Faber-Ziman partial structure factors relative to species α and β.[1] The total radial distribution functions *G(r)* are defined as

$$G(r) = \sum_{\alpha,\beta\geq\alpha}(2-\delta_{\alpha\beta})c_\alpha c_\beta b_\alpha b_\beta(g_{\alpha\beta}(r)-1) \quad (3)$$

where $g_{\alpha\beta}(r)$ are the partial distribution functions, that represent the probability density of finding an atom of species β at a distance r from an atom of species α. The *G(r)* are linked to the measured *F(Q)s* by Fourier transformation:

$$G(r) = \frac{1}{(2\pi)^3\rho_0}\int_0^\infty 4\pi Q^2 F(Q)\frac{sin(Qr)}{Qr}dQ. \quad (4)$$

From the partial distribution functions $g_{\alpha\beta}$(r), we can define a coordination number $n_{\alpha\beta}$ (r) as the number of particles β coordinated to a particle α as a function of their relative distance r

$$n_{\alpha\beta}(r) = 4\pi\rho_\beta r^2 g_{\alpha\beta}(r). \quad (5)$$

The coordination number integrated over a distance r_0, traditionally determined as the minimum of the relative *g(r)*, represents the number of molecules in the solvation shell.

Neutron total scattering data were acquired on the Near and Inter-Mediate Order Diffractometer (NIMROD) at ISIS, the UK Neutron and Muon Source, in a Q range $0.02 < Q < 50$ Å^{-1}.[2] For data correction and normalisation in absolute units, data for empty instrument, empty TiZr cans and VNb 3mm flat plate were acquired. In addition, multiple, inelastic scattering and absorption corrections were performed using the GudrunN routines.[3] Further

inelasticity corrections to the standard procedure were performed by subtracting exponential functions to the pure ionic liquid and the 0.5 molal solutions of amplitude (Table 1).[4] The introduction of exponential functions was necessary to fit the small angle regime, however the minimum Q (maximum r) accessible is constrained by he size of the simulation box. The pure ionic liquid and the 0.5 TMAF solution have sides of 48.44 Å and 60.63 Å respectively, which translates to a minimum Q of 0.13 and 0.1 $Å^{-1}$. Neutron total scattering data were acquired for 2 isotopically distinct samples, for a total of 4 samples: i) fully hydrogenated 1,3-dimethyl imidazolium (TFSI); ii) D_1 1,3-dimethyl imidazolium (TFSI); iii) 0.5 molal TMAF in fully hydrogenated 1,3-dimethyl imidazolium (TFSI); iv) 0.5 molal TMAF in D_1 1,3-dimethyl imidazolium (TFSI).

Table S 1: Amplitudes and decays of the exponential functions subtracted from the experimental data for inelasticity corrections. Note that for correcting the TMAF containing solutions, it was necessary the introduction of more exponential functions due to the higher hydrogen content.[3,4]

Data set	Amplitude	Decay
pure D_0	0.2	1.5
pure D_1	0.2	1.5
0.5 TMAF in D_0	0.5	1.5
	-0.5	0.5
	0.8	0.05
0.5 TMAF in D_1	0.5	1.6
	-0.25	1.0

Empirical Potential Structure Refinement (EPSR) modeling

Structural information was extracted from the total neutron scattering data via the Empirical Potential Structure Refinement (EPSR) method.[5] The EPSR method consists of a Monte Carlo simulation where the inter-molecular interactions between species are represented by a 12-6 Lennard-Jones potential plus a coulombic term of the form:

$$U_{inter} = U_{\alpha\beta}(r_{ij}) = 4\epsilon_{\alpha\beta}\left[\left(\frac{\sigma_{\alpha\beta}}{r_{ij}}\right)^{12} - \left(\frac{\sigma_{\alpha\beta}}{r_{ij}}\right)^{6}\right] + \frac{q_\alpha q_\beta}{4\pi\epsilon_0 r_{ij}} \tag{6}$$

where the well depth parameter $\epsilon_{\alpha\beta}$ and the range parameter $\sigma_{\alpha\beta}$ are given for a multi-component system by the Lorentz-Berthelot mixing rules[?] in terms of their values of the individual atoms

$$\epsilon_{\alpha\beta} = \sqrt{\epsilon_\alpha \epsilon_\beta}; \quad \sigma_{\alpha\beta} = \frac{1}{2}(\sigma_\alpha + \sigma_\beta). \tag{7}$$

In EPSR long range corrections to the non-Coulomb reference potential are truncated smoothly by the function

$$T(r) = \begin{cases} 1, & r < r_{minpt} \\ 0.5\Big[1 + cos\pi\Big(\frac{r - r_{minpt}}{r_{maxpt} - r_{minpt}}\Big)\Big], & r_{minpt} < r < r_{maxpt} \\ 0, & r < r_{maxpt} \end{cases} \tag{8}$$

while the Coulomb potential is truncated according Chen and Weeks:[?]

$$T_c(r) = erfc\Big(\frac{r}{\sigma_c}\Big) \tag{9}$$

where σ_c is the largest of 3.5 Å and $\frac{1}{5} r_{maxpt}$. In order to account for longer range electrostatic interaction in this system, r_{minpt} and r_{maxpt} have been extended to 15 and 18 Å. Once the simulation has equilibrated, the Empirical Potential is switched on and the Monte Carlo simulation iteratively refines the structure against the experimental neutron scattering data by adding a small empirical potential term to the reference potential, until the best agreement is reached. In this manner, the structural information is consistent with the neutron total scattering profiles of the samples.

The initial simulation was built for the pure ionic liquid by adding to the simulation box 300 1,3 dimethyl imidazolium cations and 300 TFSI anions at the atomic number density of 0.0818 atoms/Å^3. Similarly for the 0.5 molal TMAF dissolved in the ionic liquid, the simulation box was constituted of 100 TMAF molecules and 530 1,3 dimethyl imidazolium TFSI for a total of 1260 molecules. The EPSR simulation equilibrated with the Lennard Jones 'seed' potentials (Table 1) for 2000 iterations, successively the amplitude of the empirical

potential has been set to increase of 0.05 within the window of 1kT/move. Once a sufficient number of steps has been reached, the value of ereq was set up to the value that gave best agreement between the experimental and the simulated total structure factors.

In Figure 7, the total neutron scattering data are plotted against the EPSR fit for fully hydrogenated ionic liquid and for the partially deuterated imidazolium, where the most acidic proton (H_a, main text) has been selectively deuterated to obtain the necessary contrast to study its solvation behaviour. Excellent agreement is found between the experimental data and the EPSR simulations.

Atom Type	σ /Å	ϵ /kJ mol^{-1}	q /e
C1c	3.55	0.29288	-0.11
H1c	2.42	0.12552	0.21
N1c	3.25	0.71128	0.15
CMc	3.50	0.27614	-0.17
HMc	2.50	0.12552	0.13
C2c	3.55	0.29288	-0.13
H2c	2.42	0.12552	0.21
N1a	3.25	0.71128	-0.66
S1a	3.55	1.04600	1.02
O1a	2.96	0.87864	-0.53
C1a	3.50	0.27614	0.35
F1a	2.95	0.22175	-0.16
N1s	3.25	0.71128	0.12
C1s	3.50	0.27614	-0.17
H1s	2.50	0.12552	0.13
F1s	2.73	3.01250	-1.0

Figure S 1: Input Lennard-Jones Parameter and Charges for 1,3-dimethyl imidazolium$^+$ cation, TFSI$^-$ anion , TMA$^+$ cation and F$^-$ anion.

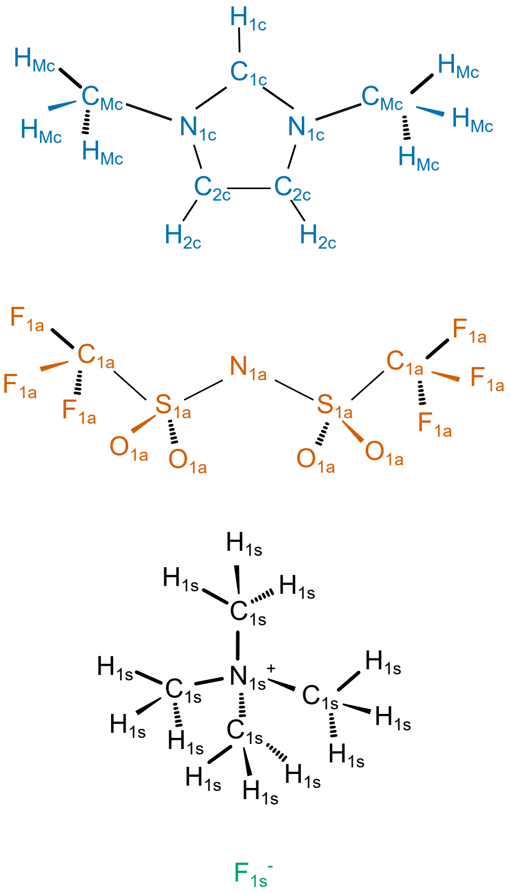

Figure S 2: Atom types as parametrised in the EPSR simulations.

Table S 2: Molecular geometries: bond lengths and bond angles for 1,3-dimethyl imidazolium^{+} cation, TFSI^{-} anion , TMA^{+} cation and F^{-} anion. Note dihedral angles are defined for half of the TFSI molecule to allow the cis and trans conformations.[6,7]

	Bond Length /Å		Bond Angle /°	Dihedral Angles /°	
C1c - H1c	1.09	C1c - N1c - C2c	108.0	C1c - N1c - C2c - C2c	0.0
N1c - C1c	1.315	C2c - N1c - CMc	125.6	C1c - N1c - C2c - H2c	180.0
N1c - CMc	1.466	C1c - N1c - CMc	126.4	CMc - N1c - C2c - C2c	180.0
CMc - HMc	1.09	N1c - C2c - H2c	122.0	CMc - N1c - C2c - H2c	0.0
N1c - C2c	1.378	C2c - C2c - H2c	130.9	C2c - N1c - C1c - N1c	0.0
C2c - H2c	1.09	N1c - C1c - N1c	109.8	C2c - N1c - C1c - H1c	180.0
		N1c - C1c - H1c	125.1	CMc - N1c - C1c - N1c	180.0
		HMc - CMc - HMc	107.8	CMc - N1c - C1c - H1c	0.0
		N1c - C2c - H2c	122.0	N1c - C2c - C2c - H2c	180.0
		C1c - N1c - CMc	126.4	N1c - C2c - C2c - N1c	0.0
		C2c - N1c - CMc	125.6	H2c - C2c - C2c - H2c	0.0
		N1c - CMc - HMc	110.7		
N1a - S1a	1.57	S1a - N1a - S1a	125.6	S1a N1a S1a C1a	-176.88
S1a - O1a	1.442	N1a - S1a - O1a	113.6	S1a N1a S1a O1a	56.77
S1a - C1a	1.86	O1a - S1a - C1a	102.6	S1a N1a S1a O1a	-83.38
C1a - F1a	1.323	S1a - C1a - F1a	111.0	N1a S1a C1a F1a	-59.43
				O1a - S1a - C1a - F1a	-59.43
				N1a - S1a - C1a - F1a	59.02
				N1a - S1a - C1a - F1a	179.38
				O1a - S1a - C1a - F1a	-59.21
				O1a - S1a - C1a - F1a	179.44
				O1a - S1a - C1a - F1a	60.48
N1s - C1s	1.494	C1s - N1s - C1s	109.47		
C1s - H1s	1.088	N1s - C1s - H1s	108.46		
		H1s - C1s - H1s	110.46		

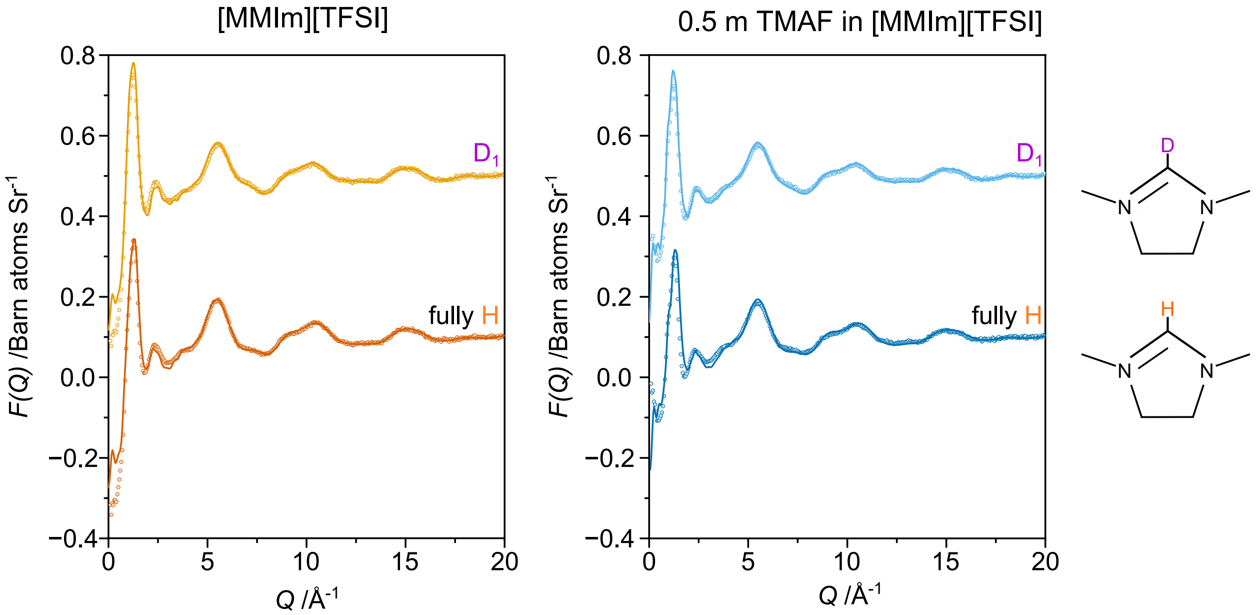

Figure S3: Neutron total structure factors F(Q) (dotted line) for the fully hydrogenated ionic liquid (orange) and the 0.5 molal TMAF solution (blue) plotted against the EPSR fit (solid line).

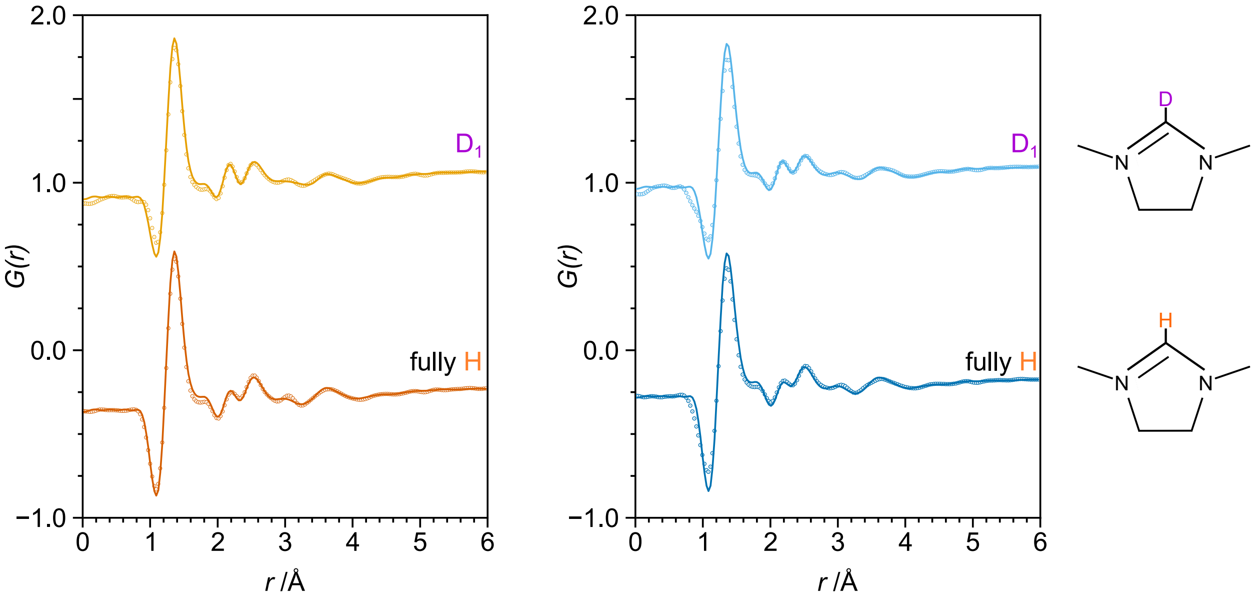

Figure S4: Neutron total pair distribution functions *G(r)* (dotted line) for the fully hydrogenated ionic liquid (orange) and the 0.5 molal TMAF solution (blue) plotted against the EPSR fit (solid line).

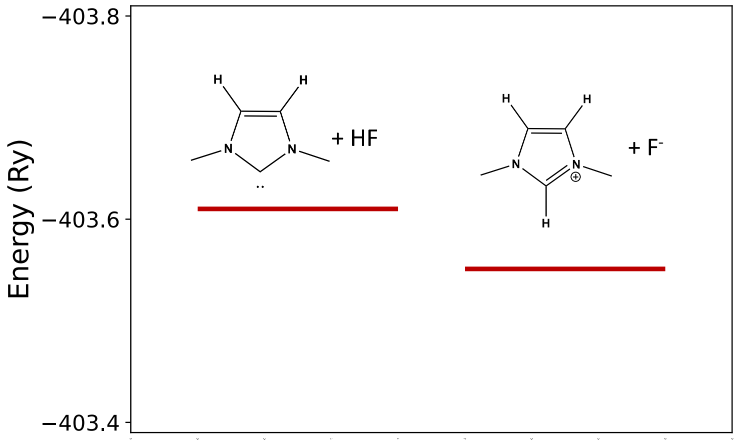

Figure S5: The ground-state energy for deprotonated imidazolium giving the carbene and the imidazolium cation.

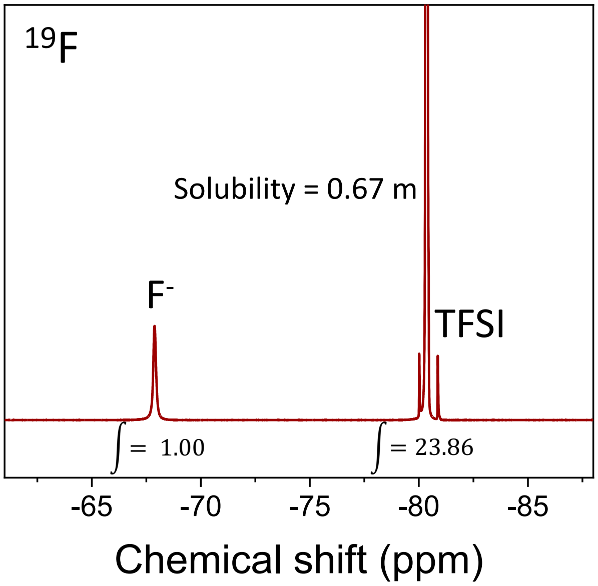

Figure S6: The fluoride solubility determined from 19F NMR.

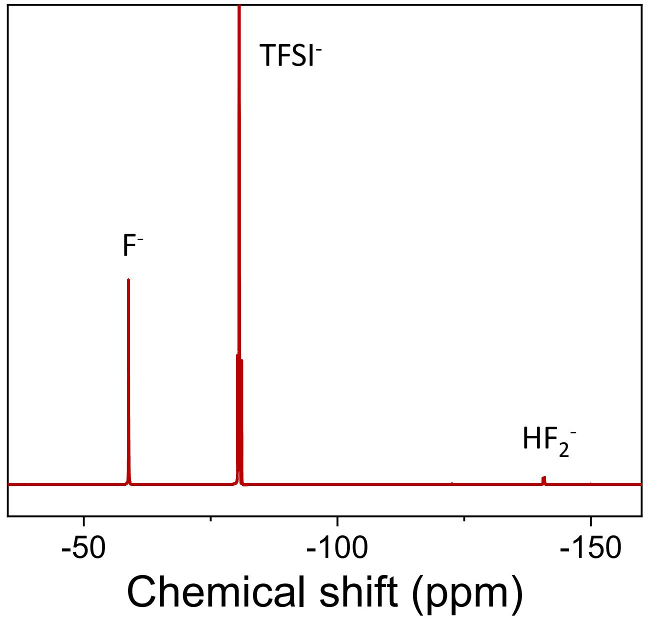

Figure S7: 0.5 m TMAF in MMIm 19F NMR spectrum showing small peak attributed to HF_2^- at -141.85 ppm.

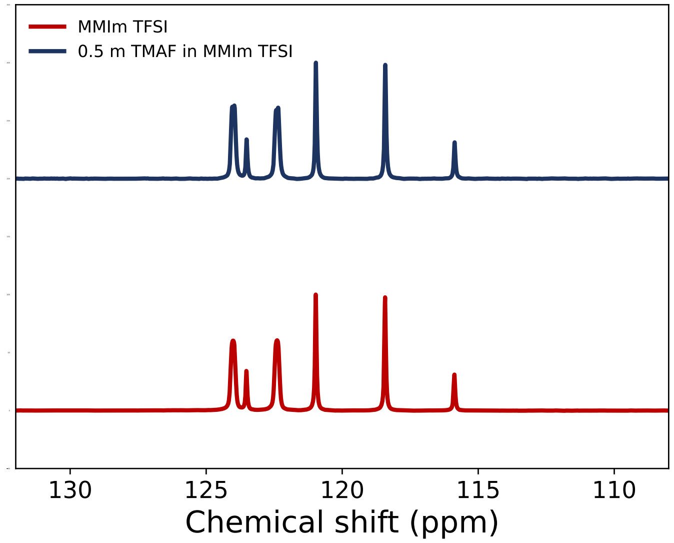

Figure S8: Enlarged ^{13}C NMR spectra for the neat MMIM TFSI and 0.1 m TMAF electrolyte showing the peak splitting in the region around 120 ppm.

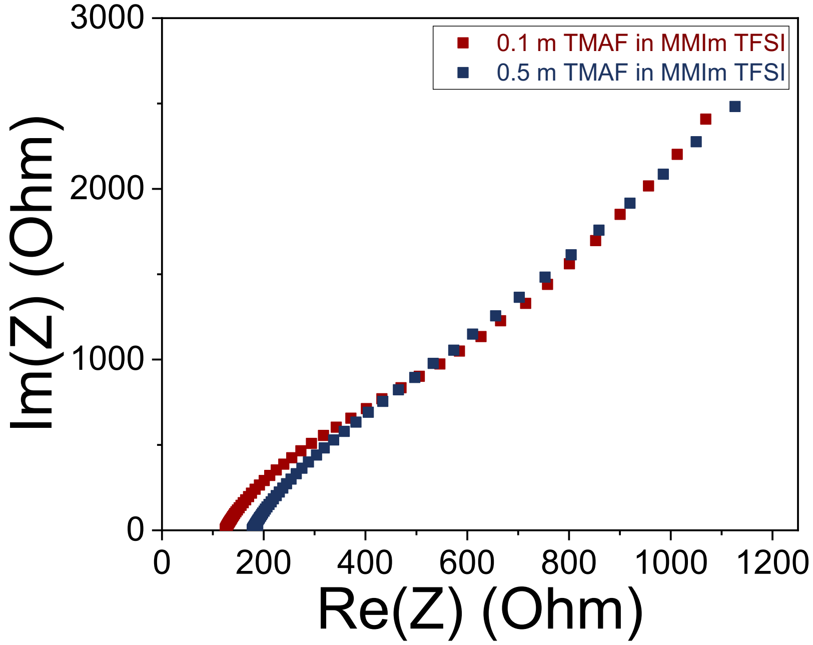

Figure S9: Raw electrochemical impedance spectroscopy data for the 0.1 m TMAF in MMIm TFSI at 30 °C for a frequency range of 1 mHz - 1 MHz.

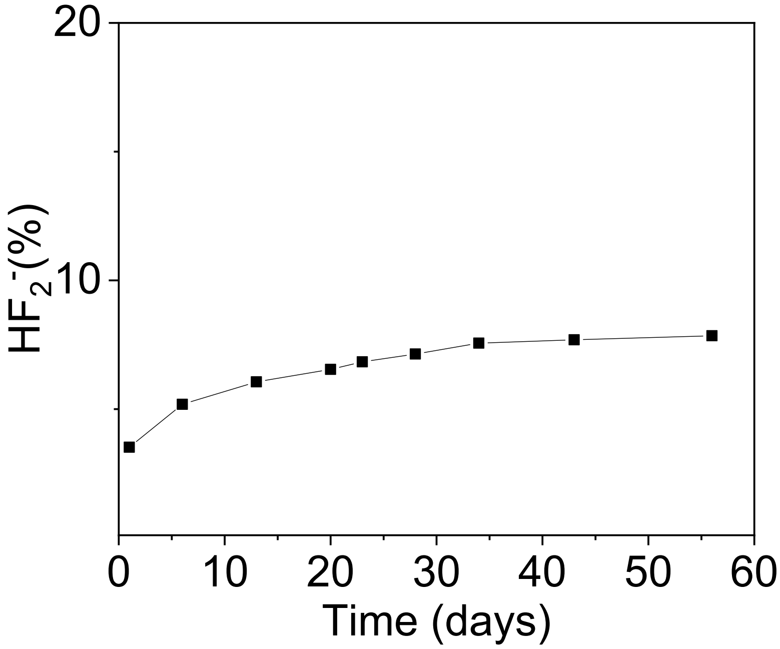

Figure S 10: HF_2^- content as measured from the ^{19}F NMR over 56 days, approaching a plateaued value of 7.8%.

References

(1) Sears, V. F. Neutron scattering lengths and cross sections. *Neutron News* **1992**, *3*, 26–37.

(2) Bowron, D. T.; Soper, A. K.; Jones, K.; Ansell, S.; Birch, S.; Norris, J.; Perrott, L.; Riedel, D.; Rhodes, N. J.; Wakefield, S. R.; Botti, A.; Ricci, M.-A.; Grazzi, F.; Zoppi, M. NIMROD: The Near and InterMediate Range Order Diffractometer of the ISIS second target station. *Rev. Sci. Instrum.* **2010**, *81*, 033905.

(3) Soper, A. K. *GudrunN and GudrunX: programs for correcting raw neutron and X-ray diffraction data to differential scattering cross section*; Science & Technology Facilities Council Swindon, UK, 2011.

(4) Soper, A. K. Inelasticity corrections for time-of-flight and fixed wavelength neutron diffraction experiments. *Mol. Phys.* **2009**, *107*, 1667–1684.

(5) Soper, A. Empirical potential Monte Carlo simulation of fluid structure. *Chem. Phys.* **1996**, *202*, 295–306.

(6) Canongia Lopes, J. N.; Pádua, A. A. H. Molecular Force Field for Ionic Liquids Composed of Triflate or Bistriflylimide Anions. *J. Phys. Chem. B* **2004**, *108*, 16893–16898.

(7) Canongia Lopes, J. N.; Deschamps, J.; Pádua, A. A. H. Modeling Ionic Liquids Using a Systematic All-Atom Force Field. *J. Phys. Chem. B* **2004**, *108*, 2038–2047.
